# LDPE and HDPE Microplastics Differently Affect the Transport of Tetracycline in Saturated Porous Media

**DOI:** 10.3390/ma14071757

**Published:** 2021-04-02

**Authors:** Enzhu Hu, Hongbo Yuan, Yichun Du, Xijuan Chen

**Affiliations:** 1 Institute of Resources and Environmental Sciences, School of Metallurgy, Northeastern University, Shenyang 110819, China; huez@smm.neu.edu.cn (E.H.); 1801652@stu.neu.edu.cn (H.Y.); 2 Shaanxi Key Laboratory of Land Consolidation, Shaanxi Provincial Land Engineering Construction Group Co., Ltd., Chang‘an University, Xi’an 710054, China; 3 Key Laboratory of Pollution Ecology and Environmental Engineering, Institute of Applied Ecology, Chinese Academy of Sciences, Shenyang 110016, China

**Keywords:** tetracycline, transport, polyethylene, microplastics, UV-weathering

## Abstract

The accumulation of microplastics (MPs) in soil and sediments may influence the penetration of contaminants into subsurface environments. However, little attention has been paid to comparing the different roles of two common polyethylene (PE) types—low-density polyethylene (LDPE) and high-density polyethylene (HDPE). In this study, the transport behaviors of tetracycline in saturated quartz sand columns in the presence and absence of these two MPs were investigated, respectively. The results showed that both types of PE MPs restrained the mobility of tetracycline at neutral conditions, while such detrimental effects were weak at acid and alkaline conditions. The degree of nonequilibrium adsorption was higher, and tetracycline transferred easier to the kinetic site for the existence of LDPE than of HDPE. The increased roughness and Brunauer-Emmett-Teller (BET) surface areas, more negative zeta potentials and the formation of oxygen function groups on the surface of MPs after UV-weathering intensified the retardation of tetracycline transport. This study revealed that the PE type and weathering should be taken into account in risk assessment, along with the solution chemistry.

## 1. Introduction

The occurrence of microplastics (MPs, <5 mm) in soil and sediment have been increasingly reported recently [1,2]. Owing to their organic characteristics, high surface area and strong hydrophobicity, MPs could adsorb many kinds of contaminants such as organic pollutants and heavy metals [3,4,5]. Tetracycline antibiotics have been extensively used for veterinary purposes in livestock and aquaculture industry for decades worldwide [6] and therefore became one of the most frequently detected emerging contaminants in soil and water [7,8].

Tetracycline could be adsorbed on many types of MPs [5], which may influence the distribution and enrichment of antibiotic resistance genes in soil and aquatic environments [9,10,11], thus intensifying their potential hazard due to the joint toxicity of both tetracycline and MPs [12,13]. The adsorption of tetracycline on MPs may also influence the penetration of tetracycline into subsurface and its migration in aquifers [14]. However, the transport behavior and mechanism of tetracycline in saturated porous media containing MPs has not been fully explored.

The adsorption of tetracycline on MPs varied with the MPs properties and environmental conditions. For instance, Yu et al. [15] reported that the adsorption amount of tetracycline on polyethylene (PE) particles was larger than those on polyvinyl chloride (PVC) and polystyrene (PS). In addition, the adsorption capacity and coefficient increased with decreasing PE particle size [15]. Moreover, Ding, et al. [16] indicated that aging could influence the surface charge, polarity, hydrophobicity, porosity, roughness and the crystallinity of MPs and thus enhance their adsorption affinity for antibiotic contaminants. In addition, the amphoteric nature of tetracycline with multiple ionizable functional groups depending on pH values would also complicate the prediction of its transport and sorption behavior [17].

PE is one of the most extensively used polymers in the world and hence has become the most commonly identified MPs in the environment [18]. According to the differences in molecular weight, crystallinity (density) and branching, PE can be divided into multiple grades with significant different physical properties, including low-density polyethylene (LDPE) and high-density polyethylene (HDPE) [19]. LDPE has a high degree of short and long side-chain branching, which results in low density and crystallinity. On the contrary, the chains of HDPE are almost linear and contain very little branching, which leads to a higher degree of crystallinity [19]. The different structures between LDPE and HDPE result in differences in their flexibility and toughness. Nonetheless, limited information is available on the differences in tetracycline adsorption between LDPE and HDPE.

In this study, four types of MPs, i.e., pristine and UV-weathered particulates of LDPE and HDPE, were mixed into quartz sand to mimic the natural environmentally relevant conditions. The transport of tetracycline through above porous media under saturated water flow condition was analyzed. The research purposes were to investigate (1) the influence of MPs on the transport of tetracycline under various solution pH conditions, (2) the different roles of LDPE and HDPE and the effect of MPs weathering on tetracycline transport.

## 2. Materials and Methods

### 2.1. Materials

The fine-grained LDPE (0.92 g·cm^−3^) and HDPE (0.95 g·cm^−3^) were purchased from Youngling Electromechanical Technology Co., Ltd. (Shanghai, China). MPs weathering experiments were carried out in a self-designed chamber (length × width × height, 48 cm × 40 cm × 30 cm) equipped with a 185 nm vacuum ultraviolet lamp (UVCN, 10-04100, 1.3 µW/cm^2^, Beijing Aerospace HONGDA Optoelectronic Technology Co., Ltd., Beijing, China). The 185 nm UV lamp generates ultraviolet energy, which is proven to effectively produce ozone and thus could accelerate the weathering process of MPs [20]. The UV-weathering experiment lasted for 2 months with 12 h radiation per day. The particle sizes of pristine and aged MPs were measured by a laser particle size analyzer (Masterizer 3000, Malvern Instruments Ltd., Malvern, Worcestershire, UK). The Brunauer-Emmett-Teller (BET) surface area of the MPs was determined by a surface area and pore size analyzer (NOVA 1200e, Quantachrome Instruments, Boynton Beach, FL, USA). The zeta (*ζ*) potentials of the pristine and UV-weathered MPs under pH values of 3, 7 and 10 were determined by a zeta potential meter (ZetaCAD, CAD Instruments, Illiers Combray France). The morphology of MPs was observed with a scanning electron microscopy (SEM, EV018, ZEISS, Oberkochen, Germany). The functional groups of pristine and UV-weathered MPs were characterized using Fourier transform infrared spectroscopy (Nicolet iS10 FTIR spectrometer, Newton Drive, Carlsbad, CA, USA). The carbon and oxygen contents of MPs were determined by an elemental analyzer (Vario Macro cube, Elementar, Langenselbold, Germany).

Quartz sands with a mean grain diameter of about 0.6 mm were purchased from Sinopharm Chemical Reagent Co. Ltd., Beijing, China. Before using, the quartz sands were purified through bake, acid soak, rinse and oven-drying following the procedure in [21].

Tetracycline (purity ≥98%) was purchased from Sinopharm Chemical Reagent Co., Ltd., Beijing, China. The stock solutions of 10 µg/mL were prepared using deionized water and adjusted to certain pH values (3, 7 and 10) using either 0.1 mol/L HCl or 0.1 mol/L NaOH. The high concentration of tetracycline was correlated with the occurrence level in the soil and manure [22]. It was set with the reference to previous column-transport studies [14,23], as well as to ensure the accurate detection and monitoring of dynamic changes in its concentrations during the experiment.

### 2.2. Transport Experiments

Borosilicate glass columns (2.74 cm inner diameter, 10.0 cm length) were dry-packed with either pure quartz sand or the mixture of quartz sand and MPs. Two pieces of 300-mesh stainless steel screens were placed on both ends of the column to prevent sand and MPs loss. Prior to packing the columns containing MPs, four types of PE particulates (pristine and UV-weathered LDPE and HDPE) were mixed thoroughly with the quartz sand, respectively. The concentration of MPs (5% *w*/*w* of MPs/sand) in the column was set according to [24,25].

After packing, the columns were first flushed with the background solution (DI water, pH of 3, 7 or 10) upwards to reach the saturation and equilibrium of the chemical condition for more than 24 h. Afterward, the tetracycline solution (10 µg/mL) was injected into the column for 450 min (5.18 pore volumes) at a constant flow rate of 0.3 mL/min (equivalent to a pore-water velocity (*v*) of 0.115 cm/min) using a peristaltic pump (BT100-2J, Longer Precision Pump Co., Ltd., Baoding, China) through PTFE pipes. The column was then flushed using the same background solution at the same flow rate (0.3 mL/min) for another 450 min.

The effluents were collected into glass tubes at a 10 min interval using a fraction collector (BS-100A, Shanghai Huxi Analysis Instrument Factory Co., Ltd., Shanghai, China). The tetracycline concentrations in the solutions were measured using a UV-visible spectrophotometer (752N, Yoke instrument Co., Ltd., Shanghai, China) at 360 nm.

All trials were performed in triplicate. The entire experimental setups, including influent container, glass column, pipes and the lid of the fraction collector were all covered by aluminum foil as much as possible to prevent any photolytic degradation of tetracycline and aging of microplastics.

The breakthrough curve (BTCs) of KBr (conservative tracer) was employed to fit the longitudinal dispersivity (*λ*) for each column using the Hydrus-1D software package (version 4.16.0110). Then, *λ* was converted to the dispersion coefficient (*D* = *λv*, cm^2^/min).

### 2.3. Mathematic Model

Previous studies have reported that the adsorption process of tetracycline on PE MPs was a chemical nonequilibrium (Yu et al., 2020). Therefore, the steady-state transports of tetracycline in the sand columns were simulated using the one-dimensional convection-dispersion equation coupled with a two-site chemical nonequilibrium sorption model, which can be found in Appendix A. Model parameters were estimated using the inverse-solution module of Hydrus-1D.

### 2.4. Breakthrough Sorption Capacity

The time for the breakthrough point (*t_b_*) was defined at the effluent concentration of 0.02*C*_0_, and the time the sorbent was considered to be essentially exhausted (*t_x_*) was chosen at the effluent concentration of 0.90*C*_0_ [26]. The time duration that tetracycline passed through the sorption zone (*t_p_*) was defined as *t_p_* = *t_x_* − *t_b_*. The value of *t_b_* and *t_x_* could be estimated using an interpolation method [27].

(1) $$t_{b}=t_{1}+\left(0.02-C_{1}\right)\times \left(t_{2}-t_{1}\right)/\left(C_{2}-C_{1}\right)$$

(2) $$t_{x}=t_{3}+\left(0.90-C_{3}\right)\times \left(t_{4}-t_{3}\right)/\left(C_{4}-C_{3}\right)$$

where *t*_1_ and *t*_2_ are time points before and after 0.02*C*_0_; *C*_1_ and *C*_2_ are concentrations before and after 0.02*C*_0_, respectively; *t*_3_ and *t*_4_ are time points before and after 0.90*C*_0_; *C*_3_ and *C*_4_ are concentrations before and after 0.90*C*_0_, respectively.

The sorption capacity between the time of breakthrough and exhaustion (*Q_c_*, µg/g) was determined by integrating the mass of tetracycline adsorbed by the unit mass of solid (quartz sand only for control, quartz sand + MPs for treatments), using a trapezoidal integration method [26].

(3) $$Q_{c}=\int_{t_{b}}^{t_{x}}\left(C_{0}-C\right)q\cdot dt/m_{s}$$

where *q* is the flow rate (mL/min); *m_s_* is the mass of adsorbent (g).

## 3. Results and Discussion

### 3.1. Characterization of PE MPs

**Figure 1:**
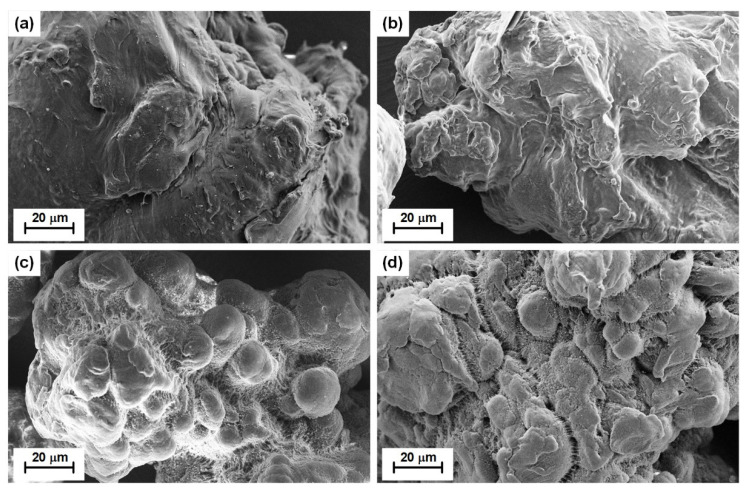
SEM images of polyethylene microplastics (PE MPs) (**a**)—pristine low-density polyethylene (LDPE); (**b**)—UV-weathered LDPE; (**c**)—pristine high-density polyethylene (HDPE); (**d**)—UV-weathered HDPE).

The surface properties of MPs characterized by SEM and FTIR were quite different between the two PE particle types. The SEM images displayed that the surface of HDPE was rougher than that of LDPE, no matter if pristine or UV-weathered (Figure 1), which caused a nearly 10–20 times larger BET specific surface area of HDPE than of LDPE (Table 1). The morphology, roughness and chemical properties of MPs surface were changed after the UV-weathering treatment.

As shown in Figure 1a, the surface morphology of pristine LDPE was relatively smooth. By comparison, microcracks were developed during the UV-weathering process (Figure 1b). In addition, some wrinkles, cracks rough and fractured flaky surface textures can be observed on the surface of UV-weathered LDPE (Figure 1b). Pristine HDPE exhibited relatively compact textures, while the UV-weathered HDPE showed a porous structure. The surface of HDPE became rougher and tended to be fragmented and cracked after UV-weathering treatment. The substantial fragmentation induced by the UV-weathering process led to an increase of the BET surface areas of MPs (Table 1). Such weathering-induced increments are more significant for HDPE than for LDPE (Table 1).

**Figure 2:**
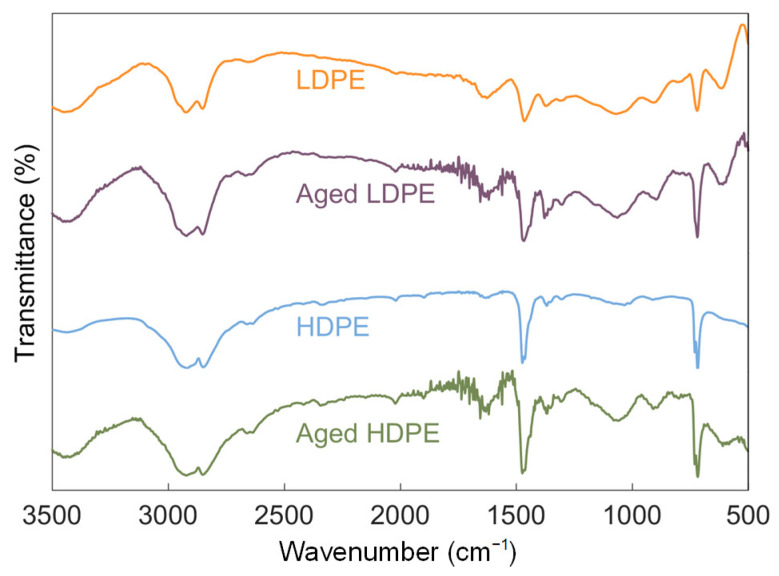
FTIR spectra of pristine and UV-weathered particulates of LDPE and HDPE.

The surface functional groups of MPs before and after UV-weathering were compared. As shown in Figure 2, the infrared spectra of LDPE and HDPE were basically the same, with abundant functional groups. The double peaks evolving at 2920 cm^−1^ and 2850 cm^−1^ are ascribed to the stretching vibrations of −CH_2_ and C-H, respectively. The bands at 908 cm^−1^ and 888 cm^−1^ corresponded to vinyl (RCH = CH_2_) and vinylidene groups (R_2_C = CH_2_), respectively [28]. The bands at 718 and 1472 cm^−1^ were ascribed to CH_2_ rocking and bending, respectively [29]. However, unless LDPE had a single broad band at each wavenumber, the FTIR spectra of HDPE showed a doublet in the frequency ranges of 1470–1463 cm^−1^ and 730–720 cm^−1^, respectively due to crystal-field splitting by chain interaction [30,31]. Of these, 1470 and 730 cm^−1^ refer to crystalline phase and 1463 and 720 cm^−1^ refer to amorphous phase [32]. After the UV-weathering treatment of MPs, variations in transmittance peak intensity can be observed in the wavenumber regions from 1700 to 1800 cm^−1^. The complex bands within this range were attributed to the stretching vibrations of carbonyl groups, including esters (1736 cm^−1^), saturated aldehyde or ketone (1719 cm^−1^) and carboxylic acids (1700 cm^−1^) [33]. These new peaks indicated MPs were oxidized due to the photo-oxidation reaction with oxygen and ozone [34]. It was also supported by the element composition analysis. The oxygen-to-carbon (O/C) atom ratio quantifying the oxidation state of MPs increased (Table 1), which indicated the formation of oxygen functional groups after UV-weathering [35].

### 3.2. Effect of pH on Tetracycline Transport

**Figure 3:**
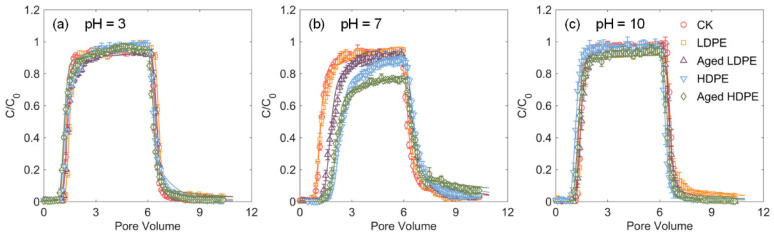
The breakthrough curves of tetracycline in pure quartz sand (CK) and quartz sand mixed with pristine and UV-weathered LDPE and pristine and UV-weathered HDPE at pH = 3 (**a**), pH = 7 (**b**) and pH = 10 (**c**), respectively.

The BTCs of tetracycline under different media and solution chemistry conditions are displayed in Figure 3. The transport parameters estimated using the two-site nonequilibrium transport model are shown in Table S1. The tetracycline transport data were well predicted with this model with R^2^ values over 0.99 (Table S1).

As shown in Figure 3, the transport of tetracycline was more inhibited at pH = 7. The breakthrough sorption capacity (*Q_c_*) reached the largest level at neutral pH. With the increasing or decreasing pH values, *Q_c_* reduced in all media types (Table S1). Tetracycline has an amphoteric molecule structure with three different functional groups that can undergo protonation-deprotonation reactions with reference to the pH value [36]. They present as a mixture of cations and zwitterions at pH = 3 with overall positive charges, as zwitterions predominantly at pH = 7 and as the pure anions at pH = 10 [17,36]. Meanwhile, the *ζ* potentials decreased gradually with increasing pH value, which showed positive values at pH = 3 and negative values at neutral and alkaline conditions (Table S1). At neutral conditions, the nonpolar surface of MPs may adsorb zwitterionic tetracycline through hydrophobic interactions [37]. In the meantime, the cation fraction may play a significant role in the overall tetracycline sorption even when the dominant species in the solution was zwitterions since the negative and positive charges of zwitterions are spatially separated and may act independently [17]. Under acidic or alkaline conditions, the sorption of tetracycline on sands or sand-MPs mixtures became restricted because of the overall electrostatic repulsion according to the same positive or negative charges between tetracycline and porous media and hence the transports of tetracycline were intensified [38].

The parameters fitted from the transport data of tetracycline in different columns showed various responses to altered solution pH (Table S1), which indicated that quartz sand, LDPE and HDPE may have different adsorption mechanisms for tetracycline. As an integrated parameter, the Damkohler number (*D_a_*) expressed the ratio of hydrodynamic residence time to characteristic time of the sorption “reaction” [39]. In the present study, *D_a_* showed a similar trend along with an increasing solution pH among treatments, which was generally greater at neutral condition than that under acid or alkaline conditions (Table S1). It was in line with previous studies that reported the adsorption of tetracycline on MPs peaked at pH 5~6 due to pH-dependent speciation of tetracycline [40,41].

### 3.3. Effect of PE Type on Tetracycline Transport

The difference on the BTCs of tetracycline was more distinct at neutral pH condition (Figure 3). Compared with the control, the presence of MPs generally increased the breakthrough sorption capacity (*Q_c_*), except the trends of CK and pristine LDPE were almost similar. The fraction of instantaneous equilibrium sites (i.e., *f* and *β*) generally increased (Table S1). In the meantime, the generally increased first-order sorption rate coefficient (*ω*) implied that tetracycline transferred easier to the kinetic site in the presence of MPs. Particularly, such a transfer was easier for LDPE than it was for HDPE according to decreasing *ω* values with the increasing PE density (Table S1). In addition, tetracycline exhibited more retarded (larger retardation coefficient, *R_d_*) BTCs in the presence of the HDPE than the LDPE (Figure 3b). The increased Damkohler number (*D_a_*) suggested that the addition of MPs into the column reduced the degree of nonequilibrium [39]. The value of *D_a_* was greater in the presence of HDPE than that of LDPE (Table S1), which implies a larger ratio of the sorption rate to the transport rate in the presence of higher density PE MPs. It was in line with the findings of [42], which reported a greater sorption velocity for LDPE than for HDPE. We need to note that the values of β and ω were less in the presence of UV-weathered HDPE than that in the control. It was probably because the aggregation of HDPE during the UV-weathering process influenced the adsorption affinity and rates of HDPE (Table 1 and Table S1).

### 3.4. Effect of PE Weathering on Tetracycline Transport

The adsorption mechanism of pollutants by MPs is more complicated after UV-weathering [43]. As shown in Table S1, UV-weathering generally reduced the ζ potentials of MPs, made them less positive at pH = 3 and more negative under both neutral and alkaline conditions. Tetracycline generally distributed more on the solid phase (higher linear distribution coefficient, *K_d_*); hence the transport was more retarded (greater retardation coefficient, *R_d_*) in the columns containing UV-weathered MPs compared with those containing pristine MPs, regardless of LDPE or HDPE. It may be because the rough surface, increased pore size and surface oxygen functional groups of MPs during the UV-weathering process controlled the ability to absorb tetracycline [43]. The parameters *ω*, *f* and *β* were generally lower in the presence of UV-weathered MPs than in the presence of pristine ones, which indicated that the nonlinearity of tetracycline adsorption by MPs increased following the UV-weathering process [43]. The adsorption of tetracycline on pristine MPs mainly related to hydrophobic interaction [16]. However, the formation of oxygen-functional groups increased the polarity, hydrophilicity and surface charges of UV-weathered MPs and thus enhanced the hydrogen bonding interaction, resulting in the tetracycline adsorbing more strongly to the surface of UV-weathered MPs [16,44,45].

## 4. Conclusions

Understanding the effect of MPs on the penetration of antibiotics into subsurface environments is of great importance since the emerging mixed contamination of antibiotics and MPs in soil and sediments has become ubiquitous. This study revealed that the presence of PE MPs would change both the sorption rate and the transport rate of tetracycline, thus affecting its migration in saturated porous media. Different PE types showed different abilities in retardation tetracycline transport, following the order of UV-weathered HDPE > pristine HDPE > UV-weathered LDPE > pristine LDPE, which was controlled by the surface characteristics of MPs. In addition, tetracycline transport was more inhibited at neutral conditions.

Findings in this work revealed that PE MPs in soil and sediments can retard the transport of tetracycline in saturated porous media. Thus, the factors of solution pH, MPs type and weathering status should be considered in risk assessment. However, such effects need to be validated for MPs of other polymer types, such as PS, PVC and polypropylene (PP) under various solution chemistry and water flow conditions in the future.

## Figures and Tables

**Table 1:** Volumetric median diameters (*D_v_*_50_), Brunauer-Emmett-Teller (BET) surface areas, oxygen-to-carbon (O/C) atom ratios of pristine and UV-weathered polyethylene MPs (mean ± SD).

Property	Pristine LDPE	UV-Weathered LDPE	Pristine HDPE	UV-Weathered HDPE
*D_v_*_50_ (µm)	46.8 ± 0.1	47.9 ± 0.5	29.7 ± 0.1	167.0 ± 1.2
BET surface area (m^2^·g^−1^)	0.31 ± 0.02	0.38 ± 0.02	3.06 ± 0.04	7.32 ± 0.10
O/C ratio	0.017 ± 0.000	0.020 ± 0.000	0.023 ± 0.000	0.030 ± 0.000

## Data Availability

Data sharing not available.

## Supplementary Materials

The following are available online at https://www.mdpi.com/article/10.3390/ma14071757/s1. Table S1: The zeta potentials of quartz sand and microplastics under different pH values, breakthrough sorption capacity of tetracycline and parameters for two-site non-equilibrium transport model as fitted using the breakthrough data of tetracycline in saturated column experiments (*n* = 3).

## Appendix A

The steady-state transport of tetracycline in sand column was simulated using the one-dimensional convection–dispersion equation coupled with a two-site chemical nonequilibrium sorption model.

(A1) $$\frac{\partial C}{\partial t}+\frac{\rho_{b}}{\theta }\frac{\partial S}{\partial t}=D\frac{\partial^{2}C}{\partial z^{2}}-v\frac{\partial C}{\partial z}$$

where *t* is the time (min); *C* is the tetracycline concentration in the aqueous phase (µg/mL); *ρ_b_* is the bulk density (g/cm^3^); *D* refers to the dispersion coefficient (cm^2^/min); *θ* is the volumetric water content (cm^3^/cm^3^); *z* is the length of domain (cm); *v* is the pore-water velocity (cm/min); *S* is the tetracycline concentration on the solid phase (µg/g), which is divided into two fractions:

(A2) $$S=S^{e}+S^{k}$$

where *S^e^* is the adsorbed tetracycline concentration on sites where sorption is assumed to be instantaneous equilibrium (µg/g); *S^k^* is the adsorbed tetracycline on kinetic sorption sites (µg/g). A linear equilibrium sorption isotherm was assumed between *S^e^* and *C*, and a linear, reversible, first-order kinetic sorption model was assumed between *S^k^* and C, such that

(A3) $$S^{e}=fK_{d}C$$

(A4) $$\frac{\partial S^{k}}{\partial t}=\omega \left[\left(1-f\right)K_{d}C-S^{k}\right]$$

where *f* is the fraction of instantaneous sorption sites assuming to be always at equilibrium with the aqueous phase; *K_d_* is the linear distribution coefficient for the two sites (cm^3^/g); *ω* is the first-order sorption rate coefficient (min^−1^).

Equations (A1)–(A4) are combined as:

(A5) $$\left(1+\frac{\rho_{b}fK_{d}}{\theta }\right)\frac{\partial C}{\partial t}+\frac{\rho_{b}\omega }{\theta }\left[\left(1-f\right)K_{d}C-S^{k}\right]=D\frac{\partial^{2}C}{\partial z^{2}}-v\frac{\partial C}{\partial z}$$

Its dimensionless form is [14]:

(A6) $$\beta R_{d}\frac{\partial c_{1}}{\partial T}+\left(1-\beta \right)R_{d}\frac{\partial c_{2}}{\partial T}=\frac{1}{P}\frac{\partial^{2}c_{1}}{\partial X^{2}}-\frac{\partial c_{1}}{\partial X}$$

(A7) $$\left(1-\beta \right)R_{d}\frac{\partial c_{2}}{\partial T}=D_{a}\left(c_{1}-c_{2}\right)$$

(A8) $$R_{d}=1+\rho_{b}K_{d}/\theta$$

(A9) $$\beta =\left(1+\rho_{b}fK_{d}/\theta \right)/R_{d}$$

(A10) $$c_{1}=C/C_{0}$$

(A11) $$c_{2}=S^{k}/\left[C_{0}K_{d}\left(1-f\right)\right]$$

(A12) T=vt/L

(A13) P=vL/D

(A14) X=z/L

(A15) $$D_{a}=\omega \left(1-\beta \right)R_{d}L/v$$

where *β* is the fraction of instantaneous retardation that related to the fraction of equilibrium sorption sites (*f*); *R_d_* is the retardation coefficient; *T* is the dimensionless time as pore volumes; *X* is the dimensionless version of axial distance; *c*_1_ and *c*_2_ are the normalized concentrations at the equilibrium site and the kinetic site, respectively; *P* refers to the Peclet number representing the dispersive-flux contribution to the convective transport; *L* is the column length (cm); *D_a_* refers to the Damkohler number expressing the ratio of hydrodynamic residence time to characteristic time of the sorption “reaction” [39].

Parameters *K_d_*, *f* and *ω* for tetracycline transport were estimated using the inverse-solution module of Hydrus-1D. Then the values of *R*, *β* and *D_a_* were calculated.
