# Supplementary material for: LDPE and HDPE Microplastics Differently Affect the Transport of Tetracycline in Saturated Porous Media
Source: Materials (Basel). 2021 Apr 2;14(7):1757. doi: 10.3390/ma14071757 (PMC8038232; doi:10.3390/ma14071757)
Supplement: Supplementary file 1 [file materials-14-01757-s001.pdf]

materials

MDPI

Supplementary Material

LDPE and HDPE Microplastics Differently Affect the Transport of Tetracycline in Saturated Porous Media

Enzhu Hu [1], Hongbo Yuan [1], Yichun Du [2,*] and Xijuan Chen [3,*]

[1] Institute of Resources and Environmental Sciences, School of Metallurgy, Northeastern University, Shenyang 110819, China; huez@smm.neu.edu.cn (E.H.); 940954685@qq.com (H.Y.)
[2] Shaanxi Key Laboratory of Land Consolidation, Xi'an 710054, China
[3] Key Laboratory of Pollution Ecology and Environmental Engineering, Institute of Applied Ecology, Chinese Academy of Sciences, Shenyang 110016, China
* Correspondence: linghuxzj@sina.com (Y.D.); chenxj@iae.ac.cn (X.C.); Tel.: +86-153-3902-1936 (Y.D.); +86-156-4026-8401 (X.C.)

Citation: Hu, E.; Yuan, H.; Du, Y; Chen, X. LDPE and HDPE Microplastics Differently Affect the Transport of Tetracycline in Saturated Porous Media. *Materials* **2021**, *14*, 1757. https://doi.org/10.3390/ma14071757

Academic Editor: Durga Parajuli

Received: 7 March 2021
Accepted: 30 March 2021
Published:2 April 2021

Publisher's Note: MDPI stays neutral with regard to jurisdictional claims in published maps and institutional affiliations.

Copyright: © 2021 by the authors. Licensee MDPI, Basel, Switzerland. This article is an open access article distributed under the terms and conditions of the Creative Commons Attribution (CC BY) license (http://creativecommons.org/licenses/by/4.0/).

Table S1. The zeta potentials of quartz sand and microplastics under different pH values, breakthrough sorption capacity of tetracycline, and parameters for two-site non-equilibrium transport model as fitted using the breakthrough data of tetracycline in saturated column experiments (n =3) [a].

Treatment	pH	ζ potential [b] (mV)	Q_c (μg/g)	D ($cm^2 \cdot min^{-1}$)	f	K_d ($cm^3 \cdot g^{-1}$)	ω	R^2	R_d	β	D_a
Sand	3	64.72 ± 0.54	0.77 ± 0.00	(0.67 ± 0.06)×10^{-2}	0.14 ± 0.04	0.65 ± 0.20	(4.6 ± 1.7) × 10^{-4}	1.00	3.30 ± 0.41	0.40 ± 0.01	0.08 ± 0.02
LDPE	3	86.18 ± 0.55	1.23 ± 0.02	(1.03 ± 0.07) × 10^{-2}	0.38 ± 0.03	0.36 ± 0.03	(1.6 ± 0.2) × 10^{-3}	1.00	2.28 ± 0.06	0.65 ± 0.01	0.11 ± 0.01
UV-weathered LDPE	3	66.33 ± 3.44	1.50 ± 0.03	(1.04 ± 0.09) × 10^{-2}	0.25 ± 0.03	0.44 ± 0.06	(1.2 ± 0.2) × 10^{-3}	0.99	2.54 ± 0.12	0.55 ± 0.01	0.12 ± 0.02
HDPE	3	70.64 ± 1.41	2.06 ± 0.01	(1.92 ± 0.14) × 10^{-2}	0.41 ± 0.03	0.14 ± 0.01	(1.3 ± 0.2) × 10^{-2}	1.00	1.49 ± 0.01	0.81 ± 0.01	0.33 ± 0.02
UV-weathered HDPE	3	38.11 ± 0.40	2.26 ± 0.02	(1.31 ± 0.12) × 10^{-2}	0.25 ± 0.02	0.21 ± 0.02	(2.7 ± 0.4) × 10^{-3}	1.00	1.74 ± 0.04	0.68 ± 0.00	0.13 ± 0.01
Sand	7	−7.33 ± 0.19	3.11 ± 0.03	(0.67 ± 0.06) × 10^{-2}	0.23 ± 0.01	0.25 ± 0.01	(3.2 ± 0.3) × 10^{-3}	1.00	1.87 ± 0.03	0.64 ± 0.00	0.19 ± 0.01
LDPE	7	−25.30 ± 0.47	3.37 ± 0.02	(1.03 ± 0.07) × 10^{-2}	0.24 ± 0.02	0.19 ± 0.01	(5.7 ± 0.6) × 10^{-3}	0.99	1.68 ± 0.02	0.69 ± 0.00	0.26 ± 0.02
UV-weathered LDPE	7	−30.32 ± 0.18	3.66 ± 0.03	(1.04 ± 0.09) × 10^{-2}	0.52 ± 0.02	0.42 ± 0.02	(4.5 ± 0.5) × 10^{-3}	0.99	2.49 ± 0.03	0.71 ± 0.01	0.28 ± 0.02
HDPE	7	−18.06 ± 0.49	4.48 ± 0.02	(1.92 ± 0.14) × 10^{-2}	0.57 ± 0.02	0.66 ± 0.02	(3.6 ± 0.3) × 10^{-3}	0.99	3.33 ± 0.04	0.70 ± 0.01	0.31 ± 0.01
UV-weathered HDPE	7	−27.83 ± 0.25	5.96 ± 0.02	(1.31 ± 0.12) × 10^{-2}	0.30 ± 0.01	1.08 ± 0.04	(1.9 ± 0.1) × 10^{-3}	0.99	4.79 ± 0.07	0.45 ± 0.01	0.43 ± 0.02
Sand	10	−100.43 ± 1.38	0.70 ± 0.01	(0.67 ± 0.06) × 10^{-2}	0.67 ± 0.02	0.15 ± 0.00	(4.6 ± 0.5) × 10^{-3}	1.00	1.53 ± 0.01	0.88 ± 0.00	0.07 ± 0.00
LDPE	10	−117.85 ± 3.32	0.93 ± 0.02	(1.03 ± 0.07) × 10^{-2}	0.38 ± 0.03	0.34 ± 0.03	(2.3 ± 0.4) × 10^{-3}	0.99	2.19 ± 0.06	0.66 ± 0.01	0.15 ± 0.01
UV-weathered LDPE	10	−144.12 ± 0.67	1.26 ± 0.01	(1.04 ± 0.09) × 10^{-2}	0.21 ± 0.05	0.62 ± 0.16	(5.3 ± 1.9) × 10^{-4}	1.00	3.18 ± 0.33	0.46 ± 0.02	0.08 ± 0.02
HDPE	10	−96.29 ± 8.64	1.64 ± 0.01	(1.92 ± 0.14) × 10^{-2}	0.24 ± 0.04	0.21 ± 0.04	(1.1 ± 0.3) × 10^{-3}	1.00	1.74 ± 0.07	0.68 ± 0.00	0.05 ± 0.01
UV-weathered HDPE	10	−118.20 ± 1.47	2.37 ± 0.01	(1.31 ± 0.12) × 10^{-2}	0.13 ± 0.03	0.70 ± 0.14	(6.0 ± 1.5) × 10^{-4}	1.00	3.47 ± 0.28	0.38 ± 0.00	0.11 ± 0.02

[a] Q_c, is the sorption capacity between the time of breakthrough and exhaustion (μg/g); f is the fraction of instantaneous sorption sites assuming to be always at equilibrium with the aqueous phase; D refers to the dispersion coefficient (cm^2/min); K_d is the linear distribution coefficient for both two sites (cm^3/g); ω is the first-order sorption rate coefficient (min^{-1}); R^2 is the coefficient of determination; β is the fraction of instantaneous retardation that related to f; R_d is the retardation coefficient; D_a is the Damkohler number.

[b] ζ potentials were measured for pure sand and PE MPs, respectively, not for the sand-MPs mixture
